# Breast Cancer and the Brain: A Comprehensive Review of Neurological Complications

**DOI:** 10.7759/cureus.48941

**Published:** 2023-11-17

**Authors:** Akshat Dubey, Suyash Agrawal, Varun Agrawal, Tanishq Dubey, Arpita Jaiswal

**Affiliations:** 1 Obstetrics and Gynaecology, Jawaharlal Nehru Medical College, Datta Meghe Institute of Higher Education and Research, Wardha, IND; 2 Medicine, Jawaharlal Nehru Medical College, Datta Meghe Institute of Higher Education and Research, Wardha, IND

**Keywords:** interdisciplinary collaboration, prognosis, quality of life, neurological complications, brain metastases, breast cancer

## Abstract

Breast cancer, one of the most prevalent malignancies globally, poses a substantial health burden with its diverse neurological complications. This comprehensive review examines the intricate landscape of breast cancer's neurological effects, encompassing brain metastases, non-metastatic complications, and their profound influence on the quality of life, prognosis, and survival of affected individuals. The mechanisms, clinical manifestations, and treatment modalities of brain metastasis and the critical role of interdisciplinary collaboration in their management are explored. Additionally, we address non-metastatic neurological complications, including paraneoplastic syndromes, treatment-related side effects, leptomeningeal carcinomatosis, and radiation-induced neurotoxicity, shedding light on the challenges they present and the importance of cognitive and emotional well-being. Prognostic factors and survival rates are discussed, emphasizing the complexity of variables impacting patient outcomes. Lastly, we underscore the vital role of collaborative care in addressing these multifaceted challenges, highlighting future research directions and the ongoing quest to enhance the quality of life for breast cancer patients.

## Introduction and background

Breast cancer is one of the most prevalent and widely recognized forms of cancer worldwide; breast cancer in men is very rare. It is characterized by the uncontrolled growth of malignant cells in the breast tissue. Breast cancer can manifest in various forms, including invasive ductal carcinoma, invasive lobular carcinoma, and clinical diagnosis, not histological subtype, among others. The World Health Organization (WHO) estimates that breast cancer is the most common cancer in women, with an estimated 2.3 million new cases diagnosed in 2020 alone. While breast cancer can be curable when detected at an early stage, it remains a significant health concern with significant morbidity [1].

Understanding the neurological complications associated with breast cancer is of paramount importance. While breast cancer primarily affects the breast tissue, it can have far-reaching consequences throughout the body, including the brain. The development of neurological complications in breast cancer patients can significantly impact their quality of life, treatment options, and prognosis [2]. Neurological complications encompass a wide range of conditions, from brain metastases to paraneoplastic syndromes and cognitive impairments resulting from cancer treatments [2]. Patients experiencing neurological complications may suffer from symptoms such as headaches, seizures, cognitive deficits, motor impairments, and psychological distress. These complications often necessitate specialized care and management strategies beyond typical oncological treatment regimens [3].

The purpose of this comprehensive review is to provide an in-depth exploration of the neurological complications associated with breast cancer. We aim to elucidate the various ways in which breast cancer can affect the nervous system, from the development of brain metastases to the less commonly discussed non-metastatic complications.

## Review

Metastatic breast cancer and brain involvement

Overview of Metastatic Breast Cancer

Metastatic breast cancer, also known as stage IV or advanced breast cancer, occurs when the cancer cells from the primary tumor in the breast spread to other parts of the body, often through the bloodstream or lymphatic system. These metastases can affect various organs and tissues, including the brain. Understanding metastatic breast cancer is crucial in the context of neurological complications, as brain metastases are a significant concern in breast cancer patients [4]. Metastatic breast cancer is characterized by its aggressive nature and can pose significant challenges in terms of treatment and management. It may manifest as a metastatic disease at the time of initial diagnosis or may occur as a recurrence following treatment for earlier stages of breast cancer [5].

Incidence and Prevalence of Brain Metastases in Breast Cancer

Brain metastases are a common complication of metastatic breast cancer. It is estimated that brain metastases occur in approximately 10-16% of patients with advanced breast cancer. This incidence can vary based on the subtype of breast cancer, with some subtypes being more prone to brain involvement than others [6]. Understanding the prevalence of brain metastases is critical for healthcare providers to monitor and manage breast cancer patients effectively, as early detection and intervention can significantly impact patient outcomes [7].

Factors Influencing Brain Metastases

A subtype of breast cancer: Breast cancer is a heterogeneous disease, and the specific subtype a patient has can significantly influence their risk of developing brain metastases. Notably, triple-negative breast cancer and human epidermal growth factor receptor 2 (HER2)-positive breast cancer subtypes have been observed to have a higher predilection for brain metastases. This suggests that the biological characteristics and behavior of cancer cells within these subtypes may make them more prone to spreading to the brain, necessitating close monitoring and targeted interventions for patients with these subtypes [8].

Hormone receptor status: The hormone receptor status of the primary breast tumor is a critical factor in predicting the likelihood of brain involvement. Tumors that are hormone receptor-positive, particularly estrogen receptor-positive (ER+), are associated with a lower risk of brain metastases than hormone receptor-negative tumors. Understanding the hormone receptor status helps guide treatment decisions and surveillance strategies for breast cancer patients [9].

Primary tumor size: The size of the primary breast tumor is another crucial determinant of the risk of brain metastasis. Larger primary tumors are often linked to an increased risk of metastasis to various sites, including the brain. This underscores the importance of early detection and treatment of the primary tumor to reduce the potential for distant spread [6].

Lymph node involvement: Lymph node involvement, as indicated by cancer cells in the lymph nodes near the breast, is a significant predictor of distant metastases, including brain involvement. This highlights the lymphatic system's role in facilitating the spread of cancer cells to various organs. It also emphasizes the importance of accurately staging breast cancer to guide treatment decisions [10-12].

Molecular and genetic factors: Breast cancer is characterized by its diverse molecular and genetic profiles. Specific genetic mutations and molecular pathways can enhance the invasive and migratory capabilities of breast cancer cells, making them more likely to invade and colonize the brain. Understanding these factors at the molecular level is crucial for developing targeted therapies that address the unique characteristics of each patient's cancer [5].

Blood-brain barrier (BBB): The BBB is the brain's natural defense mechanism that limits the passage of molecules and cells from the bloodstream into the brain. This barrier poses a challenge for cancer cells to breach, as it restricts their access to the brain. However, some subtypes of breast cancer cells have evolved mechanisms to overcome this barrier, allowing them to infiltrate the brain tissue. Understanding these mechanisms is vital for developing treatments that specifically target brain metastases while preserving the integrity of the BBB [13].

Clinical Presentation of Brain Metastases

**Figure 1:**
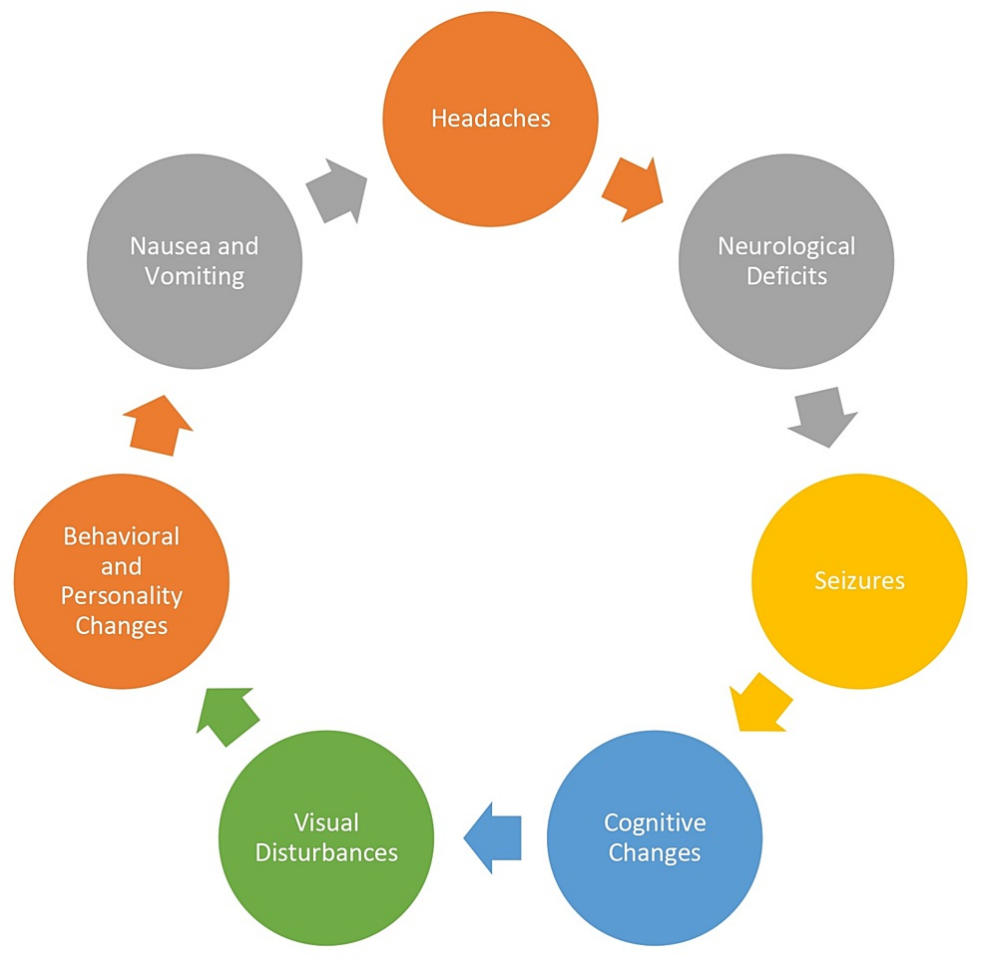
Clinical presentation of brain metastases Image Credit: Author

The clinical presentation of brain metastases in breast cancer patients can vary, but common symptoms and signs are presented in Figure 1 [11].

Mechanisms of brain metastasis

Hematogenous Spread

Hematogenous spread is one of the primary mechanisms through which breast cancer cells can reach the brain. It involves the dissemination of cancer cells through the bloodstream. Breast cancer cells can shed from the primary tumor, enter the circulation, and travel through the bloodstream to distant sites, including the brain. The process of hematogenous spread is influenced by several factors, such as the primary tumor's vascularity, tumor cell adhesion molecules, and the ability to circulate cancer cells to survive in the bloodstream [12].

Lymphatic Spread

Lymphatic spread is another route by which breast cancer cells can metastasize to the brain. While the lymphatic system primarily serves to drain lymph fluid from tissues, it can also facilitate the spread of cancer cells. Cancer cells from the primary breast tumor can enter the lymphatic vessels and travel to lymph nodes and, eventually, to other organs, including the brain. Lymphatic spread is especially relevant in cases where cancer cells reach the lymph nodes near the breast and subsequently disseminate throughout the body [10].

Infiltration Through the BBB

The BBB is a protective barrier that separates the circulating blood from the brain's neural tissue. It consists of tight junctions formed by endothelial cells in brain capillaries, which restrict the passage of molecules and cells from the bloodstream into the brain. In brain metastasis, cancer cells face the challenge of breaching this barrier. Some breast cancer cells have developed the ability to disrupt the BBB or adapt to its unique microenvironment, allowing them to infiltrate the brain tissue. Once inside the brain, these cells can establish secondary tumors [13].

Molecular and Genetic Factors

Overexpression of HER2: The HER2 is a crucial protein in cell growth and division. In some breast cancers, there is an overexpression of HER2, which can make these cancer cells more aggressive and likely to metastasize. HER2-positive breast cancers tend to grow faster and are associated with a higher risk of brain metastasis. This is because HER2 overexpression stimulates cell growth and may allow cancer cells to break away from the primary tumor and enter the bloodstream, increasing the chances of reaching the brain [14].

Molecular subtypes: Breast cancer is not a single disease but a group of diseases with distinct characteristics. Different molecular subtypes of breast cancer, such as ER+, progesterone receptor-positive, and HER2-positive, have varying propensities for metastasizing to different organs, including the brain. For example, triple-negative breast cancer, which lacks all three of these receptors, is known to have a higher risk of brain metastasis compared to other subtypes [15].

Epithelial-mesenchymal transition (EMT): EMT is a biological process through which cancer cells change their characteristics, becoming more mobile and invasive. This transition allows them to penetrate surrounding tissues and migrate to distant sites, including the brain. EMT is associated with increased aggressiveness and is thought to enhance the ability of cancer cells to form metastatic lesions by making them more adaptable to different microenvironments in the body [16].

Immune evasion: The immune system is crucial in identifying and eliminating cancer cells. However, cancer cells can develop mechanisms to evade immune surveillance, allowing them to establish metastatic lesions in the brain and other organs. By avoiding detection and attack by the immune system, these cancer cells can establish themselves in the brain and proliferate, leading to brain metastases [17].

Angiogenesis: Angiogenesis is the process by which new blood vessels are formed. Cancer cells, including those from breast cancer, can promote angiogenesis within the brain. This is significant because these new blood vessels provide essential nutrients and oxygen to the cancer cells, allowing them to thrive and grow in the brain. Developing a vascular network within the brain microenvironment is a critical step in the progression of metastatic tumors, as it provides the necessary resources for their survival and growth [18].

Clinical manifestations of brain metastases

Neurological Symptoms

**Figure 2:**
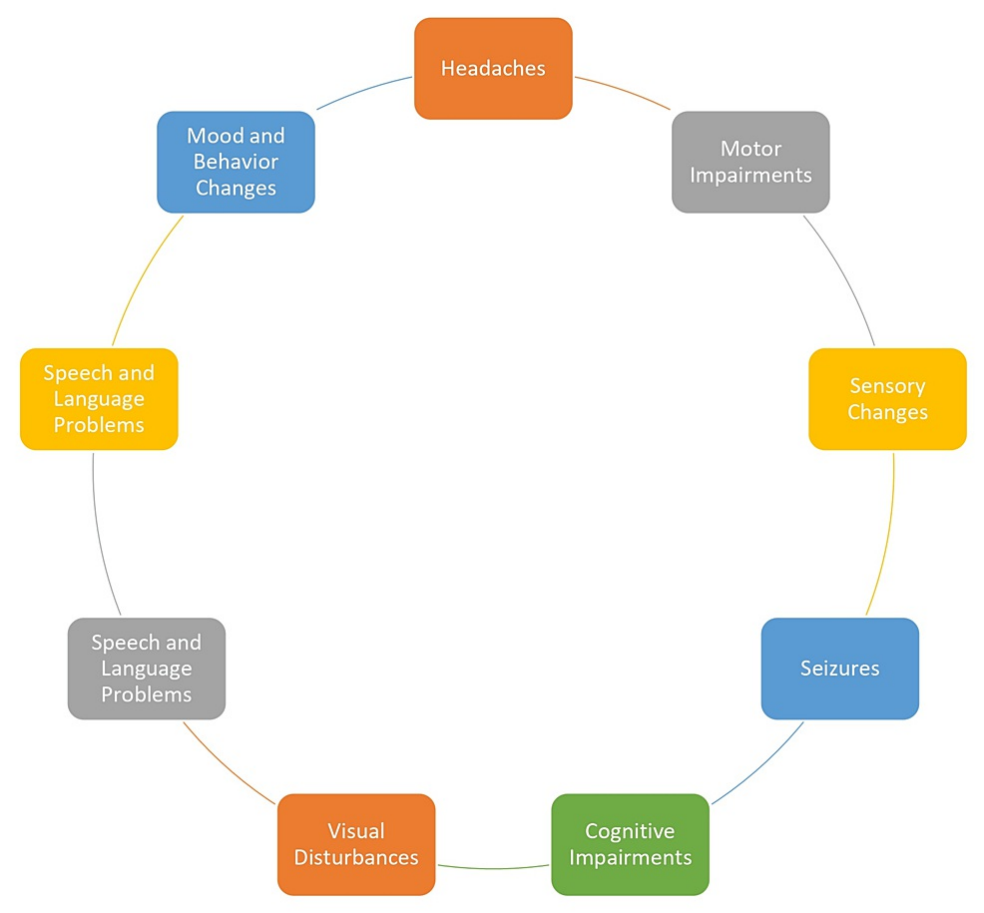
Neurological symptoms Image Credit: Author

Brain metastases often result in a range of neurological symptoms, which can vary in severity and may be influenced by the size and location of the metastatic lesions. The common neurological symptoms are described in Figure 2 [19].

Imaging Findings

Brain lesions: Radiological imaging, such as magnetic resonance imaging (MRI) or computed tomography (CT) scans, is a primary diagnostic tool for detecting the presence of brain metastases. Brain metastases typically appear as contrast-enhancing masses in these images. The contrast enhancement is due to the disruption of the BBB around the metastatic lesion, which allows contrast agents to accumulate within the tumor. These images are essential for identifying the presence of metastatic lesions in the brain, and they can help distinguish between metastatic brain tumors and primary brain tumors, which may have different treatment approaches [20].

Lesion size and location: Radiological imaging provides detailed information about brain metastatic lesions' size and precise location. This information is crucial for treatment planning. The size of the lesion affects the choice of treatment options, as larger lesions may require more aggressive interventions. Additionally, the location of the metastases in the brain is important because it determines the potential impact on neurological function and helps guide decisions about surgical resection, radiation therapy, or other therapeutic strategies. Furthermore, the number and distribution of metastatic lesions also influence treatment decisions and prognosis [21].

Peritumoral edema: Radiological imaging can reveal peritumoral edema, swelling, and inflammation in the surrounding brain tissue caused by metastatic lesions. Peritumoral edema is often seen as a zone of increased signal intensity on MR images. Understanding the extent and degree of peritumoral edema is essential because it can cause neurological symptoms and impact the patient's overall well-being. Management of peritumoral edema is a critical component of brain metastasis treatment. Physicians may use corticosteroids and other medications to reduce the edema and relieve symptoms, which can include headaches, changes in mental function, and even neurological deficits [22].

Diagnostic Methods

MRI: MRI is the gold standard for imaging the brain and detecting and characterizing brain metastases. It offers excellent soft tissue contrast and high-resolution images. MRI is beneficial for visualizing small or multiple lesions, determining their location, and assessing their relationship with surrounding brain structures. It can also help differentiate between metastatic brain tumors and primary brain tumors. Additionally, contrast-enhanced MRI can reveal the presence of contrast-enhancing masses, a hallmark of brain metastases. This imaging modality is crucial for initial diagnosis, staging, and treatment planning [23].

CT scan: While MRI is the preferred imaging method, CT scans can also be employed to identify brain metastases, especially in emergencies when MRI is not readily available or a patient cannot undergo an MRI due to medical contraindications. CT scans are faster and more readily available than MRIs and are particularly useful for detecting large, calcified, or hemorrhagic brain metastases. They can also provide important information about edema and its mass effect on surrounding brain tissue. However, compared to MRI, CT scans offer less detailed soft tissue contrast [24].

Cerebrospinal fluid (CSF) analysis: Investigating the possibility of leptomeningeal involvement is essential in cases exhibiting potential signs of dissemination in the CSF. To assess leptomeningeal metastasis, characterized by the spread of cancer to the membranes enveloping the brain and spinal cord, a comprehensive CSF analysis is conducted. This procedure entails extracting a small CSF sample via lumbar puncture (spinal tap). Subsequent examination of the CSF involves identifying cancer cells, scrutiny of cell counts for abnormalities, and detecting specific biomarkers linked to primary cancer. The diagnostic significance of CSF analysis is particularly noteworthy in cases of leptomeningeal metastasis, necessitating distinct treatment strategies compared to conventional approaches for brain metastases [25].

Neurological examination: A thorough neurological examination is a critical step in the diagnostic process. This examination, conducted by a healthcare professional, assesses the patient's clinical symptoms and neurological function. It helps identify any neurological deficits or abnormalities resulting from brain metastases. Common neurological symptoms associated with brain metastases may include headaches, changes in cognitive function, sensory or motor deficits, seizures, and behavioral alterations. The results of this examination guide treatment decisions and help monitor the patient's response to therapy [26].

Staging and Classification

The staging and classification of brain metastases are crucial for treatment planning and prognostication. Staging is often based on factors such as the number of metastatic lesions, size, and location within the brain. Classification can be further refined by considering the primary breast cancer subtype and the patient's overall health. Staging and classification systems help healthcare providers determine the appropriate treatment strategies, including surgery, radiation therapy, systemic therapy, or a combination thereof. They also assist in estimating the patient's prognosis and survival expectations, which guide discussions with the patient regarding their care and treatment options [27].

Management of Brain Metastases in Breast Cancer

The treatment options, multidisciplinary approach, palliative care, emerging treatments, and future directions for managing breast cancer patients with brain metastases are described in Table 1 [28].

**Table 1:** Treatment options, multidisciplinary approach, palliative care, and emerging treatments and future directions for managing breast cancer patients with brain metastases Table Credit: [28] WBRT: whole brain radiation therapy; SRS: stereotactic radiosurgery

Category	Description
Treatment options	Surgical intervention for single or accessible brain metastases, including craniotomy or stereotactic radiosurgery. Radiation therapy involves WBRT for multiple or widespread lesions and SRS for specific, smaller lesions. Systemic therapies include chemotherapy, hormone therapy, and targeted therapy to control the primary breast tumor and systemic disease.
Multidisciplinary approach	Involves a team of healthcare professionals, including oncologists, neurosurgeons, radiation oncologists, neurologists, and palliative care specialists. Ensures comprehensive treatment planning, individualized care, and coordinated decision-making.
Palliative care	Focuses on symptom relief, pain management, and improving the patient's quality of life. Collaborates with the primary treatment team to address the physical, emotional, and psychological needs of patients and families, providing compassionate and supportive care.
Emerging treatments and future directions	Investigational immunotherapies aim to use the immune system to target cancer cells, including those in the brain. Advancements in molecular and genetic profiles may lead to more personalized therapies. Research explores combining treatment modalities and biomarker development for early detection. Future directions may involve supportive care interventions like cognitive rehabilitation programs to address neurological deficits.

Non-metastatic neurological complications

Paraneoplastic Syndromes

Paraneoplastic syndromes are a group of rare disorders in which cancer triggers an immune response that affects distant tissues or organs, including the nervous system. In breast cancer, paraneoplastic syndromes can manifest as neurological complications and are often caused by autoantibodies produced in response to the tumor. Symptoms may include muscle weakness, difficulty with coordination, sensory changes, and cognitive deficits. Recognizing and diagnosing paraneoplastic syndromes is essential, as managing the underlying breast cancer can lead to the resolution of these neurological symptoms [29].

Side Effects of Breast Cancer Treatments

Chemotherapy-induced peripheral neuropathy (CIPN): CIPN is a recognized side effect of potent chemotherapy drugs designed to target rapidly dividing cancer cells. Beyond their anti-cancer efficacy, these drugs can also impact the peripheral nervous system, leading to the development of CIPN. This condition manifests as numbness, tingling, and pain, primarily affecting the hands and feet. The severity of CIPN symptoms varies based on factors such as the specific chemotherapy drugs employed, their dosage, and the duration of treatment [30]. To provide a more comprehensive understanding, it is imperative to delve into the specific types of neuropathy associated with chemotherapy and identify drugs that are more commonly linked to this condition. Differentiating between neuropathy types, such as sensory, motor, or autonomic neuropathy, can shed light on the diverse manifestations of CIPN. Furthermore, elucidating which chemotherapy drugs are more predisposed to induce neuropathy will contribute valuable insights into the nuances of this side effect. This information is crucial for healthcare providers in optimizing patient care and managing the potential impact of CIPN on the quality of life of individuals undergoing cancer treatment [30].

Cognitive impairment (chemo brain): Some cancer patients, particularly those who undergo chemotherapy, may experience cognitive changes often referred to as "chemo brain." Difficulties with memory, concentration, and mental clarity characterize the chemo brain. Patients may find focusing, remembering details, or performing cognitive tasks challenging. While the exact cause of chemo brain is not fully understood, it is thought to result from the combination of chemotherapy drugs, stress, fatigue, and the emotional impact of cancer diagnosis and treatment. These cognitive changes can be temporary or persist after treatment, affecting the patient's daily life [31].

Neurological effects of hormone therapy: Hormone therapy is a standard treatment for hormone receptor-positive breast cancer. Tamoxifen and aromatase inhibitors are used to block the effects of hormones that fuel cancer growth. While these therapies are generally well-tolerated, some patients may experience mood disturbances, such as depression or anxiety, as side effects. In rare cases, hormone therapy can also lead to cognitive changes, though this is less common compared to chemotherapy-induced cognitive impairment [32]. Patients need to discuss any mood changes or cognitive difficulties with their healthcare team so that appropriate interventions can be considered [32].

Radiation-induced neurotoxicity: Radiation therapy is used to target and kill cancer cells, but it can also affect nearby healthy tissues, including the brain. When radiation therapy is administered to the brain, it may result in cognitive impairment and other neurological deficits. This can occur in patients with brain metastases, where radiation is specifically targeted to the metastatic lesions, as well as in patients receiving prophylactic cranial irradiation to prevent the spread of cancer to the brain. Radiation-induced neurotoxicity can manifest as changes in memory, problem-solving abilities, and overall cognitive function. The severity and duration of these effects can vary among individuals [33].

Leptomeningeal Carcinomatosis

Leptomeningeal carcinomatosis, also known as leptomeningeal metastasis or neoplastic meningitis, represents a rare yet severe neurological complication observed in breast cancer patients [34]. This condition involves disseminating cancer cells to the meninges, the membranes surrounding the brain, and the spinal cord. A spectrum of neurological symptoms, including severe headaches, altered mental status, weakness, and cranial nerve abnormalities, marks its manifestation. The diagnosis of leptomeningeal carcinomatosis typically relies on CSF analysis and neuroimaging. While recognized as a challenging condition, it is noteworthy that intrathecal chemotherapy is not considered a standard of care despite the existence of specific protocols. Management strategies often encompass a combination of intrathecal chemotherapy and supportive care. However, it is crucial to acknowledge that the prognosis for leptomeningeal carcinomatosis is frequently poor [34]. This underlines the need for continued research and exploration of alternative therapeutic approaches to enhance the management of this complex and challenging neurological complication.

Radiation-Induced Neurotoxicity

Radiation therapy, while effective in treating breast cancer, can lead to radiation-induced neurotoxicity when administered near the central nervous system. This neurotoxicity may manifest as cognitive deficits, memory problems, and other neurological symptoms. Reducing the risk of radiation-induced neurotoxicity involves careful treatment planning, dose optimization, and minimizing exposure to sensitive brain regions. When this complication occurs, management may include supportive care, cognitive rehabilitation, and close monitoring of the patient's neurological function [35].

Impact on quality of life

Cognitive Function and Quality of Life in Breast Cancer Patients

Cognitive impairments, often referred to as "chemo brain," represent a significant concern for breast cancer patients, especially those with brain metastases or those undergoing specific cancer treatments. These cognitive deficits encompass a range of challenges, including memory problems, difficulties with concentration, and reduced processing speed. Cognitive changes may manifest as forgetfulness, difficulty organizing thoughts, and trouble multitasking. Such cognitive impairments can profoundly affect a patient's daily life, including their ability to carry out work, maintain social interactions, and sustain an overall well-being. These challenges can lead to feelings of frustration, anxiety, and a sense of reduced independence, further contributing to a diminished quality of life [36].

Recognizing and addressing cognitive changes is essential in the care of breast cancer patients to improve their overall quality of life. Supportive interventions play a crucial role in mitigating the impact of cognitive impairments. Cognitive rehabilitation programs are designed to help patients regain cognitive function and develop strategies to cope with memory and attention difficulties. These programs are tailored to the specific needs of each patient and may involve memory exercises, problem-solving techniques, and cognitive exercises to enhance mental clarity. Additionally, counseling and support groups offer patients a platform to discuss their experiences, share coping strategies, and seek emotional support. These interventions are integral components of comprehensive care for breast cancer patients, aiming to help them manage cognitive challenges and ultimately enhance their well-being as they navigate their cancer journey [37].

Psychological and Emotional Aspects

Anxiety and depression: Receiving a breast cancer diagnosis is a life-altering moment that can trigger a cascade of emotional responses. The uncertainty surrounding one's prognosis, the potential side effects of treatments, and the profound impact of the disease on various aspects of life can all contribute to heightened levels of anxiety and depression. This emotional distress is often rooted in the fear of the unknown as patients grapple with questions about their future, the effectiveness of their treatment, and the potential challenges they may face. Anxiety and depression in breast cancer patients can manifest as persistent worry, sadness, sleep disturbances, and a general sense of unease. These emotional struggles can significantly impact a patient's overall well-being and may necessitate additional support and interventions to manage them effectively [38].

Fear of recurrence: The fear of cancer recurrence is a common and potent source of psychological distress for breast cancer patients, especially those who have experienced brain metastases. Even after successful treatment, the fear of the cancer returning can persist and cast a shadow over a patient's life. This persistent fear can lead to heightened vigilance, as patients may constantly monitor their bodies for any signs or symptoms they associate with a potential recurrence. The fear of recurrence can influence a patient's decision-making process, leading to choices that prioritize cancer prevention and early detection. While these decisions can be proactive, they may also affect a patient's overall quality of life by causing chronic anxiety and stress. Patients with this fear may benefit from counseling and support to help them manage and cope with these feelings [39].

Emotional well-being: Managing the emotional aspects of breast cancer and its neurological complications is crucial for a patient's overall quality of life. Emotional well-being encompasses feelings and experiences, including hope, resilience, happiness, and peace. When emotional well-being is compromised, it can have a profound impact not only on the patient but also on their relationships and support network. Patients who are struggling emotionally may find it challenging to maintain their social connections and may withdraw from their usual activities and responsibilities. This can affect their ability to seek and receive support from loved ones, friends, and healthcare professionals. Therefore, addressing emotional well-being is essential for the patient's happiness, the preservation of their social connections, and the quality of their support system. Comprehensive care for breast cancer patients should encompass both physical and emotional aspects to ensure the best possible outcome and overall well-being [40].

Coping Strategies and Support

Coping mechanisms: Coping mechanisms are the strategies and techniques that breast cancer patients use to navigate the complex challenges they face during their cancer journey. These mechanisms are essential for managing the stress, anxiety, and emotional turmoil that often accompany a cancer diagnosis. Patients may employ various coping methods, such as seeking information about their condition and treatment options, to gain control and understanding. Additionally, many patients turn to relaxation techniques like deep breathing exercises and mindfulness to reduce stress and promote emotional well-being. Creative expression, such as art, music, or writing, can serve as a therapeutic outlet for emotions and can be a valuable means of coping with the emotional impact of breast cancer. Coping mechanisms empower patients to address the psychological aspects of their condition, fostering resilience and emotional stability [41].

Support networks: Strong support networks are invaluable for breast cancer patients. These networks can include family members, close friends, support groups, and healthcare professionals. Supportive relationships can offer emotional comfort, understanding, and a sense of belonging, which is crucial for patients dealing with the psychological and emotional challenges of breast cancer. Family and friends can provide practical assistance, such as transportation to medical appointments, help with daily tasks, and emotional support. Support groups create a safe space for patients to share their experiences, fears, and triumphs with others who can relate, reducing feelings of isolation. Healthcare professionals, including oncologists, nurses, and mental health specialists, can offer guidance, information, and medical care tailored to the patient's needs, contributing to a comprehensive support network [42].

Psychological support: Psychological support is a critical component of breast cancer care, particularly for patients grappling with anxiety, depression, and the emotional toll of their diagnosis and treatment. This support can take various forms, including individual counseling, therapy, and psychosocial interventions. Mental health professionals with expertise in oncology can work with patients to address their emotional challenges. They offer coping strategies, such as cognitive-behavioral therapy, to help patients manage anxiety and depression. Psychological support provides patients with a safe and confidential space to discuss their fears and emotions, equipping them with tools to enhance their psychological well-being and resilience throughout their cancer journey [43].

Survivorship programs: Survivorship programs are designed to assist breast cancer survivors transitioning from active treatment to post-treatment life. These programs acknowledge that the psychological and emotional impact of cancer often lingers long after treatment has ended. They offer support and resources to help survivors cope with the aftermath of their cancer experience. Survivorship programs may guide managing potential side effects of treatment, monitoring for cancer recurrence, and adopting a healthier lifestyle. These programs aim to help survivors regain a sense of normalcy in their daily lives while addressing the psychological challenges that can arise during the post-treatment phase. Overall, survivorship programs contribute to the holistic care of breast cancer survivors by addressing their physical and emotional needs [44].

Prognosis and survival

Factors Influencing Survival in Breast Cancer Patients with Brain Involvement

Stage of brain involvement: The stage of brain metastases refers to the extent and characteristics of the metastatic lesions in the brain. This includes factors such as the number of lesions, size, and location within the brain. A single, well-defined lesion that can be surgically removed often has a more favorable prognosis than multiple, inoperable lesions that may be scattered throughout the brain. The feasibility of treating brain metastases through surgery or other local therapies like radiation is a crucial consideration [21].

Primary tumor characteristics: The primary breast tumor's characteristics are significant in determining the likelihood of developing brain metastases and the overall prognosis. Breast cancer is a heterogeneous disease with different subtypes, such as hormone receptor-positive, HER2-positive, and triple-negative breast cancer. The subtype can influence the propensity of the cancer to spread to the brain. For example, HER2-positive breast cancer is more likely to metastasize to the brain. Different subtypes may also respond differently to treatments [15].

Response to treatment: How the patient responds to various treatment modalities is critical. The response to treatment can include surgical resection of the brain metastases, radiation therapy, and systemic therapies like chemotherapy, targeted therapies, and immunotherapies. Successful control of the primary breast tumor and brain metastases is associated with improved survival. In some cases, the brain metastases may be well-controlled while the primary tumor progresses, or vice versa, impacting the prognosis [21].

Performance status: The patient's overall health and performance status are essential considerations. Patients with a good performance status, meaning they have a relatively high level of physical and mental functioning and minimal comorbidities (other health issues), often have better survival outcomes. Their ability to tolerate and benefit from aggressive treatments, such as surgery and chemotherapy, is often more remarkable [45].

Molecular and genetic factors: Specific molecular and genetic factors within the breast cancer cells can significantly influence the course of the disease. For example, targetable mutations, such as mutations in the BRCA genes, may open up treatment options like poly(ADP-ribose) polymerase (PARP) inhibitors. Additionally, the expression of specific proteins, like hormone receptors or HER2, can determine the effectiveness of targeted therapies. Patients with tumors harboring these molecular features may have different treatment options and potentially better outcomes [46].

Systemic disease control: Breast cancer is not limited to the brain; it can metastasize to other distant sites in the body. The management of systemic disease, which includes controlling the primary breast cancer and other metastatic sites, is crucial for improving survival outcomes. Effective systemic treatment, which can include chemotherapy, hormonal therapy, or targeted therapies, is essential in preventing the progression of the disease in other organs and extending overall survival [47].

Survival Rates and Outcomes

Median survival: Median survival is an important statistic that indicates the point at which half of the patients diagnosed with breast cancer and brain metastases have survived longer and the other half have survived for a shorter duration. It's crucial to understand that median survival represents an average and that individual outcomes can vary widely. Factors such as the extent of brain involvement, response to treatment, and overall health play a significant role in determining where a patient falls on the survival curve. While some patients may experience a shorter survival time, others can live well beyond the median survival period [48].

One-year survival: One-year survival rates estimate the percentage of patients alive one year after the diagnosis of brain metastases. This metric is often used to assess the immediate impact of treatment and the overall prognosis. The one-year survival rate can vary based on the disease stage, the treatments' effectiveness, and the patient's specific characteristics. Patients with a favorable response to therapy and limited disease at diagnosis are more likely to achieve a higher one-year survival rate [49].

Long-term survival: Long-term survival, sometimes defined as survival beyond five years or even more, is an encouraging aspect of managing breast cancer with brain metastases. Achieving long-term survival often results from a comprehensive and individualized treatment approach. This approach may involve a combination of surgery, radiation therapy, systemic therapies, and careful management of both brain and systemic disease. It's a testament to the advances in medical care and the potential for some patients to maintain a good quality of life despite metastatic disease [48].

Follow-Up and Long-Term Monitoring

Regular imaging: Periodic brain imaging through techniques like MRI or CT scans is crucial for monitoring the status of brain metastases. These scans help healthcare providers assess brain lesions' size, number, and location. Regular imaging can reveal changes in the metastatic lesions, which may indicate disease progression or response to treatment. It is essential for making informed decisions about treatment adjustments or interventions [20].

Neurological assessment: Regular neurological assessments thoroughly evaluate the patient's neurological function, including cognitive and functional aspects. These assessments are critical for detecting neurological deficits or changes associated with brain metastases. Changes in cognitive function, motor skills, coordination, or other neurological symptoms can indicate disease progression or treatment-related side effects. Timely identification allows for appropriate interventions, including symptom management or treatment adjustments [50].

Systemic disease monitoring: While brain metastases are a significant concern, breast cancer is a systemic disease, and it can affect other organs as well. Regular monitoring of primary breast cancer and other metastatic sites, such as the bones, liver, or lungs, is essential. This ensures the disease remains well-controlled throughout the body, not just in the brain. Treatment strategies may need to be adapted based on systemic disease status to prevent further metastases and manage the primary tumor [51].

Supportive care: Supportive care is integral to a patient's overall well-being and quality of life. Patients with brain metastases may experience physical symptoms, emotional distress, and treatment-related side effects. Psychological support, pain management, and addressing side effects like nausea, fatigue, and cognitive changes are vital for enhancing a patient's comfort and quality of life. Supportive care also includes addressing the emotional and psychological aspects of living with a life-altering diagnosis.

## Conclusions

In this comprehensive review of neurological complications associated with breast cancer, we have delved into various critical aspects of this complex disease. We explored the mechanisms behind the development of brain metastases, discussed the clinical manifestations, and outlined the multifaceted approach to their management, which includes surgery, radiation therapy, and systemic treatments. Additionally, we examined non-metastatic neurological complications, such as paraneoplastic syndromes, treatment-related side effects, leptomeningeal carcinomatosis, and radiation-induced neurotoxicity, recognizing the challenges they pose to patients. The review also highlighted the profound impact of these complications on cognitive function and emotional well-being, emphasizing the need for supportive care and psychological interventions. Furthermore, we discussed factors influencing prognosis and survival, recognizing the complex interplay of clinical, pathological, and treatment-related variables that shape patients' outcomes. Finally, we stressed the importance of interdisciplinary collaboration in providing comprehensive care, addressing the challenges, and advancing future research. Future directions in breast cancer research should focus on innovative treatment strategies, predictive biomarkers, and long-term quality of life while simultaneously striving to reduce disparities and disparities in access to care. As we progress, interdisciplinary collaboration remains the cornerstone for progress in breast cancer care, providing hope for better patient outcomes and enhanced quality of life.
